# Displaced Physeal and Metaphyseal Fractures of Distal Radius in Children. Can Wire Fixation Achieve Better Outcome at Skeletal Maturity than Cast Alone?

**DOI:** 10.5704/MOJ.2007.008

**Published:** 2020-07

**Authors:** AH Syurahbil, I Munajat, EF Mohd, D Hadizie, AA Salim

**Affiliations:** Department of Orthopaedics, Universiti Sains Malaysia, Kubang Kerian, Malaysia

**Keywords:** distal radius, metaphysis, physis, wire fixation, paediatric

## Abstract

**Introduction:**

Redisplacement following fracture reduction is a known sequela during the casting period in children treated for distal radius fracture. Kirschner wire pinning can be alternatively used to maintain the reduction during fracture healing. This study was conducted to compare the outcomes at skeletal maturity of distal radius fractures in children treated with a cast alone or together with a Kirschner wire transfixation.

**Material and Methods:**

This was a retrospective study involving 57 children with metaphyseal and physeal fractures of the distal radius. There were 30 patients with metaphyseal fractures, 19 were casted, and 11 were wire transfixed. There were 27 patients with physeal fractures, 19 were treated with a cast alone, and the remaining eight underwent pinning with Kirschner wires. All were evaluated clinically, and radiologically, and their overall outcome assessed according to the scoring system, at or after skeletal maturity, at the mean follow-up of 6.5 years (3.0 to 9.0 years).

**Results:**

In the metaphysis group, patients treated with wire fixation had a restriction in wrist palmar flexion (p=0.04) compared with patients treated with a cast. There was no radiological difference between cast and wire fixation in the metaphysis group. In the physis group, restriction of motion was found in both dorsiflexion (p=0.04) and palmar flexion (p=0.01) in patients treated with wire fixation. There was a statistically significant difference in radial inclination (p=0.01) and dorsal tilt (p=0.03) between cast and wire fixation in physis group with a more increased radial inclination in wire fixation and a more dorsal tilt in patients treated with a cast. All patients were pain-free except one (5.3%) in the physis group who had only mild pain. Overall outcomes at skeletal maturity were excellent and good in all patients. Grip strength showed no statistical difference in all groups. Complications of wire fixation included radial physeal arrests, pin site infection and numbness.

**Conclusion:**

Cast and wire fixation showed excellent and good outcomes at skeletal maturity in children with previous distal radius fracture involving both metaphysis and physis. We would recommend that children who are still having at least two years of growth remaining be treated with a cast alone following a reduction unless there is a persistent unacceptable reduction warranting a wire fixation. The site of the fracture and the type of treatment have no influence on the grip strength at skeletal maturity.

## Introduction

The incidence of distal radius fractures in children was 20%-35% of all paediatric fractures^1-3^. Of these, metaphyseal and physeal fractures comprised of 20.2% and 15%, respectively^4, 5^. The associated distal ulnar fracture was approximately 56%^6^. Most of the injury occurred from falls from low-energy trauma^1, 7-9^, followed by injury at the playground or contact activities^7, 9^. Motor vehicle accident only accounted for 7.9%^7^. An undisplaced or minimally displaced fracture could be treated nonoperatively with cast immobilisation for a short period of four to six weeks^10, 11^. Displaced fracture, however, required reduction with or without wire stabilisation and immobilisation with an above-elbow cast for at least four weeks^6, 12, 13^. Redisplacement indeed was a common complication of casting alone following a satisfactory initial reduction, ranging from 7% to 39%^13-18^. Some authors recommended a primary wire fixation to maintain reduction during fracture healing, especially in cases with a high risk for redisplacement^15, 19-21^.

Little is known on the outcomes of the distal radius fractures in children specifically at or after skeletal maturity^16, 22-24^. Therefore, we conducted this study to evaluate the clinical, radiological and overall outcomes of these fractures, comparing the outcomes between cast and wire fixation at or after skeletal maturity.

## Materials and Methods

This retrospective study was conducted in a single institution at our centre from 1st November 2017 to 31st January 2019 looking into children with displaced distal radius fracture who had an initial injury at a skeletally immature age. They were treated either by cast alone or by a cast with additional K-wire fixation. The decision to treat whether with cast alone or with wire fixation and between closed and open reduction was solely dependent on the surgeon’s preference, mainly based on the degree of fracture displacement, the timing of presentation, the age of the patient, the type of fracture, closed or open fracture and the difficulty in achieving an acceptable reduction. They were immobilised for an average of 37 days after reduction. The age of assessment was at or after the skeletal maturity, which was 14 years old and above for girls and 16 years old and above for boys. Ethics approval was obtained from the Research Ethics Board of Medical Sciences at our centre.

The inclusion criteria were children having at least two years of growth remaining since the initial injury, distal radius fractures involving the physis, complete metaphyseal fractures of the distal radius, fracture treated with a cast alone, fracture treated with a cast with additional K-wire fixation, fracture treated with a closed or an open reduction and fracture associated with or without an ipsilateral ulna fracture.

The exclusion criteria were fractures treated with plate, screws, external fixation, fracture with ipsilateral neurovascular compromise, diaphyseal or proximal third radius fractures, incomplete distal radius fracture, fractures associated with ipsilateral distal radioulnar joint disruption and pathological fractures.

The metaphyseal fracture was defined as a fracture proximal and within four cm from the growth plate of the distal radius^13^. Physis fracture was defined as a fracture involving the growth plate and classified according to the Salter-Harris classification system^4^. Fracture displacement in this study was defined when the angulation of fracture was greater than 15° or when the fracture had less than 50% of bony contact or when there was a complete displacement of the fracture at the initial injury.

The data of all distal radius fractures were traced using medical and radiology records. All radiographs of the distal radius were carefully evaluated using the Picture Archiving and Communication System (PACS), which was a computerised radio-imaging system in our centre. The patients who fulfilled the above inclusion and exclusion criteria were selected. The demographic data were also recorded from the medical notes. The long-term functional and radiological outcomes were documented from the selected patients. They were asked to come to the clinic for the assessment at or after their skeletal maturity; boys, at or after the age of 16, and girls at or after the age of 14. The informed consent was obtained from patients or parents, and the patients were assessed for pain perception and the active range of motion. A radiological assessment was made of both the injured and the uninjured wrists. The grip strength was assessed for both hands.

The patients were asked regarding pain perception over the injured wrist. The pain grading system was based on Zimmermann *et al*^22^: pain-free and mild pain if it occurred at the extremes of movement and did not interfere with daily activity; moderate pain if it was sufficient to cause alteration in work of leisure activities; and severe pain if it occurred during activities of daily living or at rest^22^.

The active ranges of motion of both injured and uninjured wrists were measured and compared. The measurement included dorsiflexion, palmar flexion, radial and ulnar deviation, and supination and pronation. The measurements were taken using a hand-held Goniometer and were recorded in degrees. Restriction in the range of motion on the injured side was compared to the normal ipsilateral side and was documented. Excessive terminal motion on the injured site beyond the maximum limit of the uninjured site was not taken into account.

**Fig. 1:**
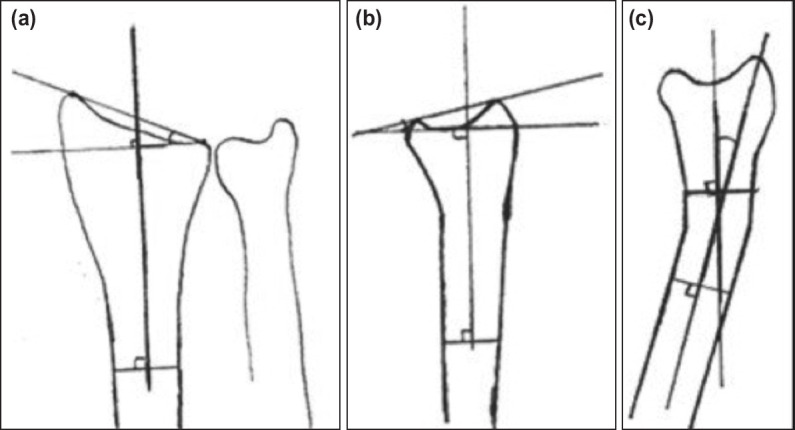
(a) An illustration showing a radial inclination which is an angle formed between a line over the plane of articular surface of the distal radius and a line perpendicular to the axis of the radius on the anteroposterior radiograph, (b) a palmar or a dorsal tilt which is an angle formed between a line over plane of articular surface of distal radius and a line perpendicular to the axis of the radius on the lateral radiograph, (c) a dorsal or a palmar angulation which is an angle formed between axis of proximal and distal parts of fracture fragments.

The standard anteroposterior and lateral radiographs of the bilateral wrists were taken during the visit. The measurements for radial inclination, palmar or dorsal tilt and dorsal or palmar angulation were performed using the measurement tools in the PACS system. They were in accordance with the measurement method by Zimmermann *et al*^22^ (Fig. 1).

**Fig. 2:**
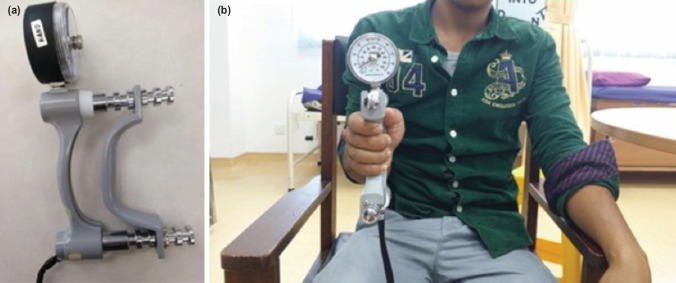
(a) A photo showing a JAMAR hydraulic hand-held Dynamometer used in our study, (b) a photo demonstrating on how the measurement of grip strength was performed by using that device.

The grip strength of both hands was measured using the JAMAR^®^ hydraulic hand-held dynamometer [Sammons Preston, Inc.] (Fig. 2a). Techniques of measurement were in accordance with the method described by Kamarul *et al*^25^. Patients were seated comfortably on the chair with shoulder adducted, elbow flexed to 90°, forearm and wrist in neutral position (Fig. 2b).

Patients were instructed to start with the injured hand grasping the dynamometer holder in a single movement and then resting for five seconds. This set of movement was repeated for the uninjured side. A total of six sets was performed for each side. The mean of three sets was taken and measured in kilograms (kg). The ratio from the study by Kamarul *et al*^25^ was applied in the analysis to account for hand dominance and gender. For right-hand dominance, a factor of 0.12 larger than left hand was taken for both men and women. For left-hand dominance, grip strength for the right hand was smaller than the left hand with a factor of 0.03 for men and a factor of 0.06 for women^25^. The final value for the grip strength was then calibrated accordingly before performing a statistical comparison.

The measurements of the active range of motion were conducted with two independent examiners during the clinic visit to verify the reliability using the inter-class correlation coefficients (ICC). A single examiner conducted the grip strength and the radiological measurement throughout the study period.

The long-term assessments were recorded as overall results in accordance with the scoring system proposed by Zimmermann *et al*^22^. The parameters used in the scoring system included pain, restriction of active range of motion and radiological angulation. The scores were graded into excellent 3-4 points, good 5-6 points, moderate 7-8 points and poor 9-12 points^22^, as shown in Table I.

**Table I:** Scoring system for overall results as suggested by Zimmermann *et al*^22^

Parameters	Points
**Pain**	
Pain-free	1 point
Mild	2 points
Moderate	3 points
Severe	4 points
**Active Range of motion**	
Equal	1 point
<10%	2 points
10-25%	3 points
>25%	4 points
**Radiogram**	
Equal	1 point
Angulation <10 degree	2 points
Angulation 10-25 degree	3 points
Angulation >25 degree	4 points
**Overall results**	
Excellent	3-4 points
Good	5-6 points
Moderate	7-8 points
Poor	9-12 points

All statistical analyses were conducted using SPSS (Statistical Program for Social Sciences) version 24.0. Non-parametric analyses were used because the sample size was relatively small and was not normally distributed. The comparisons between each group for restriction of range of motion, radiological parameters and grip strength were performed using Mann Whitney-U test. The categorical data for pain perception was compared using the Fisher Exact test. The p-value < 0.05 was considered as statistically significant. The inter-observer variability was analysed using the intra-class coefficient correlation.

## Results:

Between the years 2009 and 2014, there were a total of 57 patients with displaced distal radius fracture of a skeletally immature age. They fulfilled the criteria for this study and returned to the clinic for a long-term assessment. Informed consents were obtained from patients and parents at the clinic visit. Table II showed the demographic details of the enrolled patients. There were 30 patients (52.6%) in the metaphyseal group, 19 (63.3%) of whom had cast immobilisation alone, while 11 (36.7%) had an additional Kirschner wire inserted. In the physis group, there were 27 patients (47.4%), 19 (70.4%) who had cast immobilisation alone, and eight (29.6%) who had a Kirschner wire as a supplementary to maintain the reduction. The patients had an initial injury at the mean age of 11.4 years (range: 6.0 – 13.0 years) and returned for the final long-term assessment at the mean age of 18.3 years (range: 15.0 – 21.0 years). The mean of the follow-up duration was 6.5 years (range: 3.0 – 9.0 years) after the initial injury.

**Table II:** Demographic details of the patients and their proportions

	Total number (n)	Percentage (%)
Total	57	100
**Gender**		
Male	49	86.0
Female	8	14.0
**Fracture Type**		
Metaphysis	30	52.6
Physis	27	47.4
Intervention		
Cast	38	66.7
Wire	19	33.3
Metaphysis		
Cast	19	63.3
Wire	11	36.7
Physis		
Cast	19	70.4
Wire	8	29.6
**Injured Side**		
Right	23	40.4
Left	33	57.9
Both	1	1.8
**Hand Dominant**		
Right	53	93.0
Left	4	7.0
Age at Initial Injury		
Below 10	5	8.8
Above 10	52	91.2
**Associated ipsilateral ulna fracture**		
Yes	26	45.6
No	31	54.4
**Open fractures**		
Yes	1	1.7
No	56	98.3

n=total number

In terms of pain perception at the final assessment, there was no statistically significant difference between the interventions and the type of fractures (Table III). All patients were pain-free over the wrist except for one from the physis group (5.3%) who had mild wrist pain (p=0.70).

**Table III:** Comparison in pain perception between cast and wire in both metaphysis and physis groups

Variables	n	Pain perception		p-value^a,b^
		Free (%)	Mild (%)	
**Metaphysis**				
Cast	19	19 (100)	0	NA
Wire	11	12 (100)	0	
Total	30			
**Physis**				
Cast	19	18 (94.7)	1 (5.3)	0.70
Wire	8	8 (100)	0 (0)	
Total		27		

^a^ Fisher Exact Test

^b^ p<0.05 was considered as statistically significant

NA, not applicable due to constant value for pain perception

The inter-class coefficient correlation for the measurement of active range of motion showed good reliability (0.76 - 0.97). In terms of restriction of the active range of motion of the wrist and forearm over the injured side compared to the non-injured side (Table IV), there was a statistically significant difference in palmar flexion (p=0.04) in the metaphysis group, and both dorsiflexion (p=0.04) and palmar flexion (p=0.01) in the physis group. Patients who had the wire in the metaphysis group had a restriction in palmar flexion, but those who had the wire in the physis group had a restriction in both dorsiflexion and palmar flexion. However, there was no statistically significant difference found in the rotation of the forearm for both the cast and the wire in the metaphysis and physis group (p=0.07 and p=0.52, respectively).

**Table IV:** Comparison of restriction in active ROM between cast and wire in metaphysis and physis groups

Parameters	Metaphysis						Physis			
	Total n=30	Cast n=19	Wire n=11	Mann Whitney U Test		Total n=27	Cast n=19	Wire n=8	Mann Whitney U Test	
	Median	Interquartile Range				Median	Interquartile Range			
Restriction in Active ROM (difference in degree compared to normal contralateral side)		25th	75th	Z	p-value^§^		25th	75th	Z	p-value^§^
Dorsiflexion	0.00	0.00	2.25	-0.474	0.64	2.00	0.00	4.00	-2.105	0.04*
Palmarflexion	0.00	0.00	2.75	-2.048	0.04*	0.00	0.00	2.00	-2.763	0.01*
Radial Deviation	0.00	0.00	2.50	-1.309	0.19	0.00	0.00	0.00	-0.129	0.90
Ulnar Deviation	0.00	0.00	2.00	-1.544	0.12	0.00	0.00	3.00	-1.698	0.09
Supination	0.00	0.00	0.00	-0.761	0.45	0.00	0.00	0.00	0.00	1.00
Pronation	0.00	0.00	0.00	-1.314	0.19	0.00	0.00	0.00	-0.649	0.52

ROM, range of motion; n, number of patients

^§^p<0.05 was considered as statistically significant

The evaluation of radiological parameters showed no statistically significant difference in the metaphysis group for both cast and wire (Table V). However, in the physis group, there was a statistically significant difference in radial inclination (p=0.01) and dorsal tilt (p=0.03) between cast and wire. An increase in radial inclination was encountered more in patients treated with wire fixation, whereas there was more dorsal tilt found in patients treated with a cast.

**Table V:** Comparison of radiological parameters between cast and wire in metaphysis and physis groups

Parameters	Metaphysis						Physis			
	Total n=28	Cast n=17	Wire n=11	Mann Whitney U Test		Total n=27	Cast n=19	Wire n=8	Mann Whitney U Test	
	Median	Interquartile Range				Median	Interquartile Range			
Radiological (difference in degree compared to normal contralateral side)		25th	75th	Z	p-value^§^		25th	75th	Z	p-value^§^
Radial Inclination	0.20	-0.35	1.00	-1.037	0.30	1.10	-0.70	2.40	-2.603	0.01*
Palmar Tilt	1.65	0.03	1.65	-.0659	0.51	0.60	-0.30	3.40	-1.966	0.05
Dorsal Tilt	0.00	0.00	0.00	-0.307	0.76	0.00	0.00	0.00	-2.221	0.03*
Dorsal Angulation	0.00	0.00	0.00	-1.091	0.28	0.00	0.00	0.00	-1.632	0.10
Palmar Angulation	0.00	0.00	0.00	-0.193	0.85	0.00	0.00	0.00	-1.512	0.13

n, number of patients

^§^ p<0.05 was considered as statistically significant

Assessment of grip strength revealed that there was no statistically significant difference between the cast and the wire fixation in both metaphysis and physis groups after taking into consideration hand dominance and gender (Table VI).

**Table VI:** Grip strength at skeletal maturity between cast and wire in metaphysis and physis groups

Grip strength	Type of intervention	N	Median	IQR	Z	p-value^i,j^
Metaphysis	Cast	19	0.99	(0.91-1.06)	-0.872	0.58
	Wire	11				
	Total	30				
Physis	Cast	19	1.03	(0.90-1.14)	-0.717	0.47
	Wire	8				
		Total	27				

IQR, Interquartile Range

^i^ Mann Whitney-U Test

^j^ p value <0.05 is considered as statistically significant

In terms of overall results, both the metaphysis and the physis group showed excellent and good overall results (Table VII). In the metaphysis group, those treated with the cast had excellent overall results of 94.1% and good in 5.9%, whereas those treated with the wire had excellent in 90.9% and good in 9.1%. In the physis group, all patients with the cast alone had 100% excellent overall results, whereas those treated with the wire had excellent overall results of 75% and good in 25%.

**Table VII:** Overall outcomes at skeletal maturity according to scoring by Zimmermann *et al*^22^

		Metaphysis		Physis	
	N(%)	Cast (%)	Wire (%)	Cast (%)	Wire (%)
Overall results	n=55 (96.5%)*	n=17 (100)	n=11 (100)	n=19 (100)	n=8 (100)
Excellent		16 (94.1)	10 (90.9)	19 (100)	6 (75)
Good		1 (5.9)	1 (9.1)	0 (0)	2 (25)
Moderate		0 (0)	0 (0)	0 (0)	0 (0)
Poor		0 (0)	0 (0)	0 (0)	0 (0)

* the overall results did not include 2 patients in metaphysis group treated with cast who refused radiograph

Almost all patients treated with the cast alone underwent manual reduction except in three cases in which the fractures were displaced as defined, but the surgeon preferred not to do any manual reduction. However, these three cases still showed good outcome at skeletal maturity.

In this study, the complications of distal radius fractures were pin site infection (5%), nonspecific mild numbness over the hand (5%), radial physeal arrest (21%) and ulnar physeal arrest (5%). There were no cases of vascular complication and osteomyelitis.

**Fig. 3:**
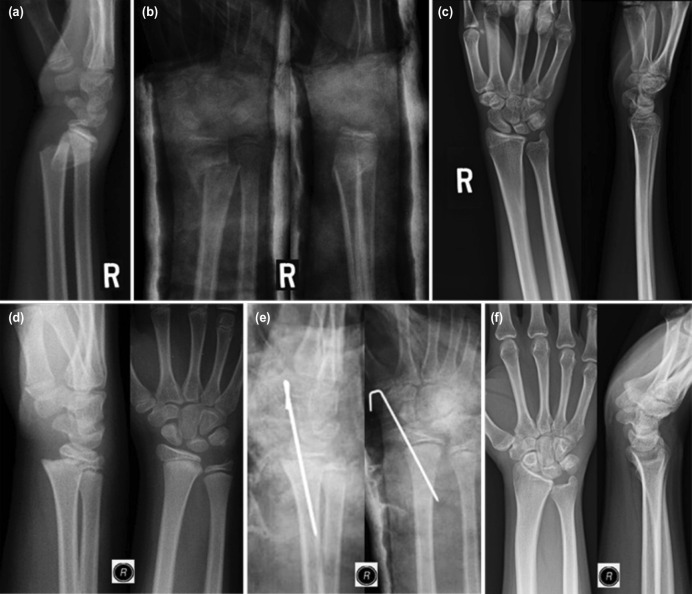
(a) Photos showing the pre-reduction lateral radiograph of a metaphyseal fracture of the distal right radius with dorsal angulation about 45° in a 13-year-old boy, (b) immediate reduction of the fracture followed by a cast application improved the dorsal angulation on lateral radiograph and showed good bone contact on AP view, (c) good remodeling and bone union as seen at skeletal maturity, (d) distal physeal fracture of the right radius on a different child who was a 12-year-old boy with dorsal translation and dorsal tilt of the epiphysis, (e) closed reduction and percutaneous smooth K wire corrected the displacement and secured the reduction, (f) and finally AP and lateral radiographs at skeletal maturity revealed complete healing of the fracture without evidence of physeal arrest.

**Fig. 4:**
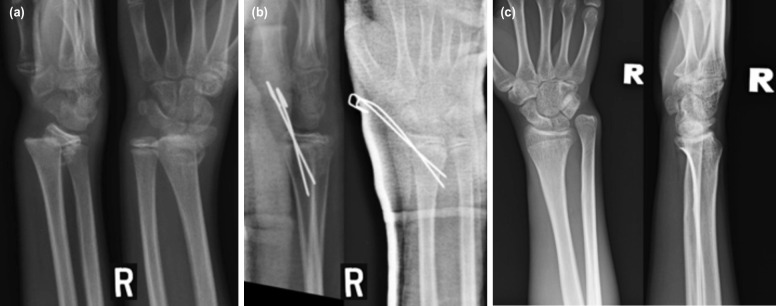
Photo showing (a) the initial fracture of the distal radial physis with dorsal displacement and angulation in one of the children in this study who was a 12-year-old boy, (b) the physeal fracture was successfully reduced both on AP and lateral views, and was immediately fixed with two smooth K wires to maintain the reduction, and (c) at skeletal maturity, the physeal arrest of the distal radius was observed on plain radiographs manifested by reduction in radial height compared to the ulna on AP view and loss of normal metaphyseal contour of the distal radius subarticularly on lateral view. The metaphysis immediately under the wrist joint appeared short and deformed on lateral radiograph. Despite this, the child had good overall result at skeletal maturity.

The fracture configurations pre-reduction, post-reduction and the final bone union at skeletal maturity are illustrated in Fig. 3 using metaphyseal fracture treated with a cast alone and physeal fracture treated with K-wire as case illustrations. The radial physeal arrest observed radiographically at skeletal maturity in distal physeal fracture treated with K wire are illustrated in Fig. 4.

## Discussion

Displaced distal radius fracture in children, usually requires initial reduction and a short period of cast immobilisation^10, 11^. However, the loss of initial reduction is the most common complication during the casting period^13-18^. Thus, several authors recommend wire fixation at initial injury in fractures with a high risk of redisplacement to ensure that the reduction is well maintained during fracture healing^15, 19-21^. On the other hand, some authors accept more angulation up to 20°-30° in patients aged less than 9-10 years without the need for a secondary manipulation with wire stabilisation, with acceptable functional outcomes^15, 26, 27^. Remodelling continues to occur as long as the physis is still open. Any injury near or at growth plate requires a final assessment at or after skeletal maturity age, particularly in those who have a fracture near to skeletal maturity age in which the remodelling capacity becomes less predictable^28^. To our knowledge, there are only a few reports in the medical literature on the long-term functional and radiological outcomes of fractures at, or after, skeletal maturity, particularly comparing between the usage of the cast alone and the cast with a wire fixation in these fractures^16, 22-24^.

In our study, we included only patients who had an initial injury at least two years before reaching skeletal maturity age. Girls and boys are expected to reach skeletal maturity at the age of 14 and 16 years old, respectively^29^. With at least two years of growth remaining, the residual angulation up to 20°, was still expected to remodel adequately, based on remodelling speeds in the published studies^21, 30, 31^. Friberg *et al*^30^, showed that the exponential pattern of radial correction was 0.9° per month in dorsal-palmar direction and 0.8° per month in radio-ulnar direction. Nietosvaara *et al*^21^, also demonstrated a remodelling speed of 1° to 2.7° per month in his series. This finding was also supported by Jeroense *et al*^31^, who showed an overall average rate of correction of 2.5° per month.

Now the question is, when should we consider using a Kirschner wire in distal radius fracture in children? Previously published literature suggested a wire fixation for the fractures with a high risk of redisplacement^15, 19-21^. Choi *et al*^20^, performed an immediate wire fixation in an age less than 16 years with a high risk for redisplacement, where there was a loss of contact of more than 50% between fragments, and found that only 9 (6.4%) of 140 children had lost their reduction^20^. Similarly, Van Leemput *et al*^12^ and Hang *et al*^32^, recommended primary wire fixation in unstable distal radius fracture, with a complete initial redisplacement and an associated distal ulnar fracture. However, Luscombe *et al*^33^, evaluated their institutional protocol for selective wiring for unstable displaced distal radius fracture and found that wire fixation did not alter the rate of redisplacement and secondary manipulation. Mani *et al*^17^, also demonstrated that radial translation carried significant risk factor for redisplacement and advocated immediate wire fixation in cases of radial translation of more than 50%. In our study, failure after reduction with complete fracture displacement was among the indications for a wire fixation.

We believe that fracture with complete displacement initially and fracture with loss of more than 50% contact in between the fragments are those with a high risk of displacement and these fractures warrant wire fixation if closed reduction with the cast fails. Repeated attempts of reduction in these high-risk fractures may damage the growth plate partially or even completely. The wire fixation itself, of course, will not guarantee to prevent redisplacement in all high-risk cases, but we strongly believe that the wire fixation will minimise the rate of redisplacement and help to maintain the reduction while the fracture is uniting. Knowing that the remodelling potential is good especially in children, the proper initial reduction must still be carried out rather than leaving totally for the remodelling process to realign and to recontour the fracture without any attempt at reduction. Good initial reduction is still important as it will ensure that the fracture will heal in proper alignment and angulation without any visible deformity or functional limitation that the patient and the parents have to face over months or years. We can take full advantage of the remodelling process that the children have, to gradually correct the deformity after a fracture. However, depending solely on the remodelling process without any attempt at a reduction is unwise since the fracture may not have complete remodelling in some instances, leaving the deformity persistent until adulthood.

The acceptable degree of angulation after fracture reduction still varied in previous literature. Do *et al*^34^, accepted angulation less than 15° in any direction and shortening less 1cm as it subsequently achieved complete remodelling without functional limitations. Mani *et al*^17^, also agreed that angulation exceeding 15° regardless of direction or bayonetting fragments was not acceptable. In a long-term study by Zimmermann *et al*^22^, they found that angulation more than 20° or apposition less than 50% between fragments had the worst functional outcomes. However, in those studies, they did not mention age factors in term of the remodelling potential. Angulation of less than 30° was accepted by Roth *et al*^27^, in an age less than nine years and by Planka *et al*^26^, in an age less than twelve years. In our study, four cases in the cast group had reangulation of more than 20° during the casting period, but they did not undergo second remanipulation and wire stabilisation. At skeletal maturity, these four patients had no or minimal restriction of motion as compared to the wire fixation group. In our study, the majority of patients had wire fixation when the angulation was more than 20°. There were only two cases with angulation less than 20° that had wire fixation.

We agree with Zimmerman *et al*^22^, that angulation of 20° is the maximum limit for acceptability in distal radius fracture. However, we limit this angulation for those who are still having two years of growth remaining which is evident in our series that all four children with angulation around 20° post-reduction did well after skeletal maturity. For children younger than ten years old with more years of growth remaining and remodelling, we accept the angulation up to 30°, which is in accordance with the study published by Roth *et al*^27^, previously.

Complications of distal radius fractures in our study include radial physeal arrest (21%, four cases), associated ulnar physeal arrest (5%, one case), pin site infection (5%, one case) and numbness (5%, one case). In previous literature, radial physeal arrest was a rare complication, ranging from 1% to 7%^21, 23^. In our series, the rate of radial physeal arrest was higher in comparison to the reported incidence. Four of our distal radius physeal arrests initially had Salter-Harris type II fracture at the time of injury. The distal ulna physeal arrest was documented to have a metaphyseal fracture, but it was later treated with wire fixation. We think that the higher incidence of growth arrest in our study was attributed to two cases of open physeal fractures. Both cases were complicated by physeal arrest. Other reported complications in previous literature included wire migrations into bone (5%) and out of bone (1%), hypergranulation of the wound (5%), infected wound (3%), ulnar nerve neuropraxia (1%) pin site infection (5.7%), superficial radial nerve injury in one case and extensor tendon problem in one case^9, 13, 20^.

For overall results, the majority of the patients in our study had excellent and good outcomes in both metaphysis and physis groups regardless of whether they were treated with a cast alone or with additional wire fixation. Although the findings showed discrete limitation in dorsiflexion, palmar flexion, altered radial inclination and dorsal tilt, the activities of daily living were not significantly affected in our study. A similar finding was also noted in a retrospective study on Salter-Harris type II fracture distal radius with a mean follow-up of 35.5 years by Cannata *et al*^23^, in which they found that none of the patients reviewed at follow-up, complained of any symptom related to their previous injury, not even those engaged in heavy manual labour. Another study by Ramoutar *et al*^9^, in his short-term retrospective review of 248 metaphyseal distal radius fracture, revealed that 87% had no functional deficit, but 10% had mild, 2% had moderate, and 1% had a severe functional limitation. They also noted that the functional limitation was attributed to the residual angulation exceeding 15°, as compared to a group with less than 15° of residual angulation9. However, their mean follow-up was only 6.6 weeks in which the residual angulation exceeding 15° might have not completely remodelled, thus producing functional limitation still at that particular time.

In our study, the small numbers of patients with fractures involving the physis, and treated with the wire resulted in difficulties in the analysis. However, reports in the literature have small sample numbers as well, when reporting fractures involving the physis treated with wire, reflecting on the difficulty in recruiting the cases^21, 33^. Nietosvaara *et al*^21^, performed percutaneous pin fixation only in 5 out of 109 children who had physeal fracture of the distal radius. Luscombe *et al*^33^, also, achieved a perfect fracture reduction in all children who sustained Salter-Harris II injury, and these patients required neither remanipulation nor percutaneous wire fixation. We also found that patients were reluctant to return to the clinic a few years after the initial injury since they did not complain of limitation during daily activities.

Surgical training for proper fracture reduction and insertion of wire should be emphasised to prevent the incidence of physeal arrest. The long-term outcomes at skeletal maturity are essential in aiding decision making for surgical intervention. It also helps in counselling anxious parents who are concerned with apparent deformity of the wrist because of the residual angulation.

Studies specifically looking into the grip strength following the distal radius fracture in children are still few. Houshian *et al*^24^, measured the grip strength of both hands in Salter-Harris type II epiphyseal plate injury of the distal radius using Martin’s Vigrometer (Germany) with a median follow-up of 8.5 years and reported that the grip strength was normal in all 85 patients. Similarly, Roth *et al*^27^, studied 66 distal metaphyseal forearm fractures in children and found that after a mean of four years, all had a full grip strength and all had returned to normal activities without restrictions. These findings were consistent with our study, which showed no difference in the grip strength between both hands after a mean follow-up of 6.4 years. Cannata *et al*^23^, found four of 139 (2.9%) Salter-Harris type II distal radius fractures, who were followed for an average of 25.5 years where there was an associated decreased grip strength. This study had the longest follow-up on the long-term outcome of the grip strength following the distal radius fractures in children.

## Conclusion

Cast and wire fixation show excellent and good outcomes at skeletal maturity in children with previous distal radius fracture involving both metaphysis and physis. We would recommend that children who are still having at least two years of growth remaining can still be treated with a cast alone following a reduction unless unacceptable reduction persists warranting wire fixation. The site of the fracture and the type of treatment have no influence on the grip strength at skeletal maturity. Despite discrete functional and radiological differences, no limitation was seen in the activities of daily living at the final follow-up in our study.
